# A combination of antibodies against Bm86 and Subolesin inhibits engorgement of *Rhipicephalus australis* (formerly *Rhipicephalus microplus*) larvae *in vitro*

**DOI:** 10.1186/s13071-019-3616-3

**Published:** 2019-07-25

**Authors:** Jos J. A. Trentelman, Hendry Teunissen, Jos A. G. M. Kleuskens, Jos van de Crommert, José de la Fuente, Joppe W. R. Hovius, Theo P. M. Schetters

**Affiliations:** 1 Center for Experimental and Molecular Medicine, Amsterdam Infection & Immunity, Amsterdam UMC, Univ of Amsterdam, Meibergdreef 9, 1105AZ Amsterdam, The Netherlands; 2 Mérieux NutriSciences, Pascalstraat 25, 6716 AZ Ede, The Netherlands; 3 MSD Animal Health, Wim de Körverstraat 35, 5830 AA Boxmeer, The Netherlands; 4 Aduro Biotech Europe, Kloosterstraat 9, 1101 RX Boxmeer, The Netherlands; 5 SaBio. Instituto de Investigación en Recursos Cinegéticos IREC (CSIC-UCLM-JCCM), 13005 Ciudad Real, Spain; 6 Department of Veterinary Pathobiology, Center for Veterinary Health Sciences, Oklahoma State University, Stillwater, OK 74078 USA; 7 ProtActivity, Sering 36, 5432 DD Cuijk, The Netherlands; 8 Department of Veterinary Tropical Diseases, University of Pretoria, Onderstepoort, 0110 South Africa

**Keywords:** Artificial tick feeding, *In vitro* screening, *R. microplus*, *R. australis*, Subolesin, Bm86, Vaccine

## Abstract

**Background:**

*Rhipicephalus microplus* is a hard tick species that has a high impact on cattle health and production in tropical and subtropical regions. Recently, ribosomal DNA and morphological analysis resulted in the reinstatement of *R. australis* as a separate species from *R. microplus*. Both feed on cattle and can transmit bovine pathogens such as *Anaplasma* and *Babesia* species. The current treatment with acaricides is becoming increasingly less effective due to the emergence of resistant tick strains. A promising alternative can be found in the form of anti-tick vaccines. The available commercial vaccines can be used to control tick infestation, but the lack of a knockdown effect (> 90% reduction in tick numbers as seen with effective acaricides) hampers its widespread use, hence higher efficacious vaccines are needed. Instead of searching for new protective antigens, we investigated the efficacy of vaccines that contain more than one (partially) protective antigen. For screening vaccine formulations, a previously developed *in vitro* feeding assay was used in which *R. australis* larvae are fed sera that were raised against the candidate vaccine antigens. In the present study, the efficacy of the Bm86 midgut antigen and the cytosolic Subolesin (SUB) antigen were evaluated *in vitro*.

**Results:**

Antiserum against recombinant Bm86 (rBm86) partially inhibited larval engorgement, whereas antiserum against recombinant SUB (rSUB) did not have any effect on feeding of larvae. Importantly, when larvae were fed a combination of antiserum against rBm86 and rSUB, a synergistic effect on significantly reducing larval infestations was found. Immunohistochemical analysis revealed that the rBm86 antiserum reacted with gut epithelium of *R. australis* larvae, whereas the antiserum against rSUB stained salivary glands and rectal sac epithelium.

**Conclusions:**

Combining anti-Bm86 and anti-subolesin antibodies synergistically reduced *R. australis* larval feeding *in vitro*. *Rhipicephalus australis* is a one host tick, meaning that the larvae develop to nymphs and subsequently adults on the same host. Hence, this protective effect could be even more pronounced when larvae are used for infestation of vaccinated cattle, as the antibodies could then affect all three developmental stages. This will be tested in future *in vivo* experiments.

## Background

*Rhipicephalus microplus* is a hard tick that has a major impact on cattle health in tropical and subtropical regions. Tick attachment and feeding on cattle has a direct negative effect on cattle production [1]. Recently, ribosomal DNA and morphological analysis resulted in the reinstatement of *R. australis* as a separate species from *R. microplus* [2]. Next to production loss through feeding, ticks can transmit a range of diseases, including anaplasmosis and babesiosis. It is therefore of great importance to control tick infestations to ensure livestock health, productivity and the livelihood of rural smallholder communities. To date, tick control heavily depends on the use of tick-resistant breeds and treatment of susceptible breeds with acaricides, but tick resistance to these acaricides is becoming problematic [3].

An alternative to acaricide treatment could be vaccination with tick antigens. Early studies have shown that vaccination with crude tick antigen preparations was indeed able to induce antibodies and interfere with feeding and subsequent further development, thereby reducing tick infestation [4]. However, preparation of crude tick extracts is cumbersome and not feasible for the development of a commercial anti-tick vaccine. With the arrival of recombinant protein techniques, single protein antigens could be evaluated for protective activity. This led to the commercial and industrial production of Bm86, a tick midgut antigen first described in 1989 [5] which forms the basis of two commercial anti-tick vaccines (Gavac^TM^, Heber Biotech; TickGard, Merck Animal Health) [6, 7]. The efficacy of these vaccines in the field was estimated on average 55% reduction of the number of engorged adult female *R. microplus* ticks, which hampers its widespread use [6].

After the discovery of Bm86 and its success as the first recombinant anti-tick vaccine, numerous studies have been performed, identifying multiple tick antigens as reviewed previously [8, 9]. From these reviewed antigens, Ribosomal protein P0 was shown to have the highest overall efficacy of 96% [10]. However, as experimental vaccination studies with these antigens showed a maximal reduction of 70% on the number of engorged female adults, none of these appear to be a vast improvement over the current Bm86-based commercial vaccines nor approaching the efficacy of acaricides. Vaccination with partially purified tick extracts increased protection against *R. microplus* compared to Bm86 alone, indicating that the effect of Bm86-based vaccines could be increased through the addition of other tick antigens [11]. Hence, it seems more feasible to build on Bm86-based vaccines and increase their anti-tick efficacy by optimizing their formulation.

A more recently described antigen is Subolesin (SUB). It was discovered in 2003 through cDNA Expression Library Immunization of an *I. scapularis* derived IDE8 embryonic cell line and subsequent Expressed Sequence Tag analysis [12] under the name of 4D8, later renamed Subolesin [13]. Phylogenetic analysis showed that SUB is an orthologue of Akirin [14]. Akirin is involved in the innate immune response of *Drosophila melanogaster* and is thought to function as a transcription factor in NF-κB dependent gene expression in insects and mammals [15]. Knock out of the SUB gene in RNAi experiments showed degeneration of salivary glands, midgut and reproductive organs [13]. RNAi of SUB showed a negative effect on tick weight and high reduction in tick progeny after feeding for multiple hard tick species [13, 16]. It has also been shown that RNAi with SUB reduced *R. microplus* infestation rate, tick weight and oviposition, whereas vaccination only affected *R. microplus* infestation rate and oviposition [17, 18].

Currently, vaccination-challenge trials in cattle are being used to evaluate and select *R. microplus* and *R. australis* candidate vaccine antigens. However, these experiments are costly, time consuming and are under limitation due to their negative impact on animal welfare. An *in vitro* feeding model for *R. australis* is an attractive alternative to evaluate the anti-tick effect of immune sera. There have been multiple reports of the use of *in vitro* feeding for ticks in literature [19–31]. There are two methods to feed ticks *in vitro*; capillary feeding and membrane feeding. Using capillary feeding, ticks are fed to repletion by placing capillary tubes over the hypostome [19–21]. This technique is limited to the feeding of semi-engorged adult ticks as the ticks mouthparts need to be large enough to fit the capillary tube and ticks have to be very eager to imbibe blood. Membrane feeding tries to mimic the natural situation where ticks attach to either animal skin or an artificial membrane [22–25]. Depending on the membrane used, membrane feeding can be used for larval and nymphal ticks as well [26–28]. In both systems, ticks are fed naïve bovine blood (defibrinated or supplemented with anti-coagulants) and when *in vitro* efficacy of tick antigens are tested, specific antibodies or anti-serum was added. It is known that natural tick immunity consists of both humoral and cellular immune components. However, it can be partially transferred by antibodies alone. This led Evin & Kemp to postulate that an anti-tick vaccine should consist of antigens that can be targeted by antibodies and the formation of the antibody-antigen complex should disrupt vector biology [32–34]. As defibrinated blood comes from naïve animals and the specific anti-serum does not contain immune cells, any observed anti-tick effect *in vitro* would, therefore, be highly antibody mediated. Consequently, any observed anti-tick effect with *in vitro* feeding could be an underestimation of an anti-tick effect *in vivo* as cellular and humoral immune components interplay *in vivo*. *In vitro* feeding of larvae with small hypostomes as *R. microplus* through an artificial membrane proved to be highly challenging. Only recently we described the development of an *in vitro* feeding system for *R. australis* larvae that can be used to evaluate the inhibitory activity of antisera against tick antigens [35]. Here we evaluated the *in vitro* effect of antisera against Bm86, antisera against SUB, and combinations thereof on engorgement of *R. australis* larvae.

## Methods

### Tick larvae

Tick larvae were obtained from a colony of *R. australis* that was routinely passaged on Holstein calves (Merck Animal Health Innovation GmbH, Schwabenheim, Germany). Fully engorged female ticks were collected from the calves and allowed to oviposit in Petri dishes. The resulting egg-masses were collected in laboratory tubes and allowed to hatch at 22 °C and 90% humidity. Four to six-week-old *R. microplus* larvae were used in the feeding experiments.

### Tick naïve bovine serum

For the production of normal serum, blood from healthy tick naïve Holstein Friesian cattle was collected in BD Vacutainer® Plus plastic serum tubes. Blood was allowed to clot for 1 h at 37 °C, centrifuged for 15 min at 1000×*g*, serum was removed and stored at − 20 °C.

### Antigen production, vaccination and serum collection

**Fig. 1:**
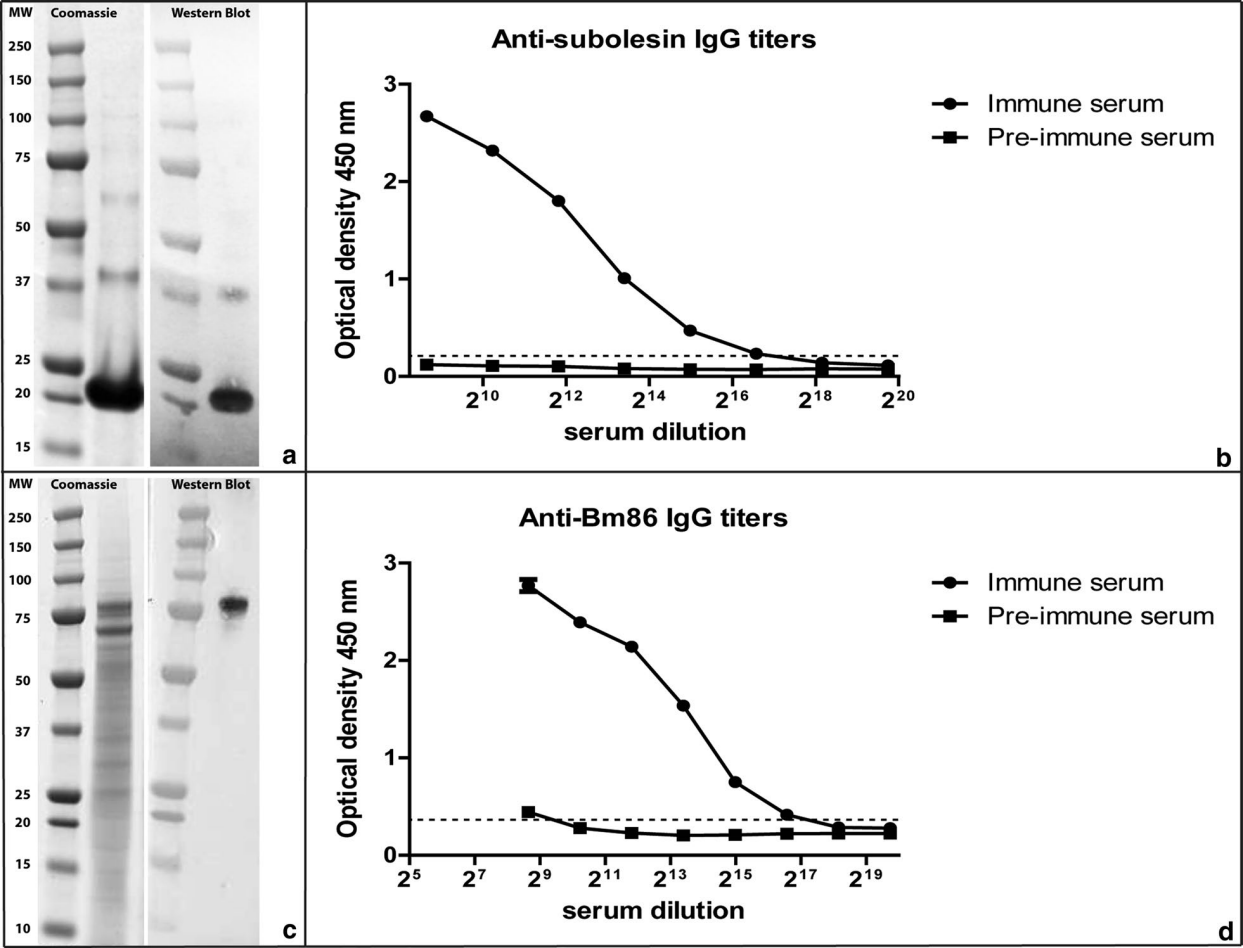
Recombinant Bm86 and subolesin and determination of antigen specific antibody titers. **a** Coomassie staining (left) and Western blot using anti-HIS mouse IgG (right) of purified subolesin (SUB) using a 4–20% Bis-Tris gel. **b** Anti-SUB antibody titration by sandwich ELISA; recombinant SUB was captured with anti-HIS mouse IgG and pooled SUB vaccinated cow serum was diluted to calculate endpoint titers. End-point titer cut-off (Bmin*2) is indicated by dashed line. **c** Coomassie staining (left) and Western blot using Bm86 (*P. pastoris-*produced) specific rabbit IgG (right) of recombinant Bm86. **d** Anti-Bm86 antibody titration by sandwich ELISA adapted from Trentelman et al. [35]. Bm86 (baculovirus produced) was captured with Bm86 (*P. pastoris-*produced) specific rabbit IgG and pooled SUB vaccinated cow serum was diluted to calculate endpoint titers. End point titer cut-off (Bmin*2) is indicated by dashed line

Recombinant Bm86 was produced in the *Baculovirus* expression system as described before [35]. Recombinant SUB was produced in *E. coli*, inclusion bodies denatured with 6M Ureum buffer and SUB was subsequently purified using a HIS-trap column (Profinia IMAC cartridge, Biorad, California, USA) and dialysed against 50 mM MES buffer (Fig. 1). Production of recombinant protein was confirmed by western blot with rabbit anti-rBm86 (*Pichia pastoris-*produced) antiserum (1:100) and with a mouse monoclonal antibody (MSD Animal Health, Boxmeer, Netherlands) against poly-histidine (His_6_-tail) for isolated rSUB-HIS_6_. Antigen-specific bovine serum was produced as follows: five Friesian Holstein cattle were subcutaneously vaccinated 2 times at a 3-week interval in the neck region with either Bm86 or SUB in water in oil adjuvant (Montanide ISA 50V2, Seppic, Paris, France).

Two weeks after the last vaccination with each antigen, blood was collected for serum production. Serum was pooled before feeding and antibody reactivity was quantified through ELISA (see below).

### Bm86 ELISA

Anti-Bm86 bovine serum titers were tested in a sandwich ELISA. In short, purified IgG from rabbit anti-rBm86 (*Pichia pastoris-*produced) antiserum (5 µg/ml in bicarbonate/carbonate coating buffer) was coated overnight on a Greiner F ELISA plate at room temperature. The wells were subsequently blocked for 1 h with 200 µl/well 1% w/v bovine serum albumin (BSA) in 0.04 M isotonic PBS at 37 °C. Next, *Baculovirus-*produced rBm86 was added to the plate (0.12 µg/ml in 1% w/v BSA in EIA-tween80 buffer, 100 µl/well) and left to incubate for 2 h at 37 °C. Vaccinated cattle serum was diluted [in 1% w/v BSA in GLD/1 buffer supplemented with 10% (v/v) naïve dog serum] and 100 µl/well subsequently added to the plate for 1 h incubation at 37 °C. Goat anti-bovine IgG-HRP (Jackson ImmunoResearch Inc., Westgrove, USA) was 2500 times diluted in 1% w/v BSA in EIA-tween80 buffer and 100 µl/well added to incubate for 1 h at 37 °C. Finally, 100 µl/well substrate (185 µl TMB and 1 ml UP-buffer in 10 ml water for injection) was added and left to incubate for 15 min in the dark at room temperature. The reaction was stopped with 50 µl/well 4N H_2_SO_4_ and OD was measured at 450 nm. Antibody titres were calculated as end point titres (Cut off is Bmin*2) using the ABend Vertical CBA v2.29 software package (MSD animal Health, Boxmeer, Netherlands).

### SUB ELISA

Antibody titres against rSUB-HIS_6_ recombinant antigen were determined using a sandwich ELISA. Briefly, Greiner F ELISA-plates were coated overnight with a mouse monoclonal antibody (MSD Animal Health, Boxmeer, Netherlands) against poly-histidine (His_6_-tail). Plates were washed and blocked with 1% w/v BSA in 0.04 M isotonic PBS and a standard amount of rSUB-His_6_ antigen in EIA-tween 80 was added to the plates. After incubation three-fold serial dilutions of serum samples in 1% w/v BSA and 10% v/v naïve dog serum in GLD1 buffer was added. Next, total bound immunoglobulin antibodies were detected by incubation with a secondary antibody goat-anti-bovine IgG conjugated with peroxidase. Finally, 100 µl/well substrate (185 µl TMB and 1 ml UP-buffer in 10 ml water for injection) was added and left to incubate for 15 min in the dark at room temperature. The reaction was stopped with 50 µl/well 4N H_2_SO_4_ and OD was measured at 450 nm. Antibody titres were calculated as end point titres (Cut off is Bmin*2) using the ABend Vertical CBA v2.29 software package (MSD Animal Health).

### Immunohistochemistry

Unfed *R. australis* larvae were fixed with 4% v/v formaldehyde, later dehydrated for 30 min at each of the following ethanol concentrations, 70%, 80%, 90% and 100% v/v, routinely embedded in paraffin wax and 3–5 µm sections were made. Hematoxylin and eosin staining was performed following routine histological procedures. Sections used for labeling with anti-rSUB antibodies were blocked with 1% w/v BSA, incubated with anti-rSUB rabbit serum (1:400). Naïve rabbit serum (1:400) was used as a control. Bm86 antigen detection was performed by labeling with anti-rBm86 antibodies after pre-incubation of the thin sections with proteinase K for 30 min. Slides were subsequently blocked with 1% w/v BSA, incubated with anti-rBm86 rabbit serum (1:400). As a control naïve rabbit serum (1:400) was used.

### Artificial tick feeding

The feeding units were used as described before [35]. Feeding membranes were made from baudruche membranes of less than 30 µm thickness (Preservation Equipment Ltd, Diss, United Kingdom) treated with silicone to add strength and flexibility. Silicone mixture was prepared: 15 g Wacker silicone E4, 9 g Silicone oil AP 200 (Sigma-Aldrich, St. Louis, Missouri, United States) and 5.8 g Hexane. After carefully mixing, 1.5 mg silicone mixture per cm^2^ was applied with a gloss paint roller. The siliconized membrane was left to polymerize overnight at room conditions. Final membrane thickness was measured with a micrometer. Membranes with a maximal thickness of 40 µm were used for feeding.

The feeding membrane was clamped in the feeding unit and 75 µl methanol bovine hair extract was added to each well and left to dry for 30 minutes at room temperature in order to apply bovine scent to the siliconized side of the feeding membrane. Next, the unit was turned upside down and *R. australis* larvae were added to the wells (approximately 100 larvae per well). Netting was used to cover the plate and the lower plate was immediately mounted using the bolts to contain the larvae. The unit was then put upright, which stimulated contact between the serum and the larvae; as a result of their questing behavior larvae crawled up to the underside of the membrane.

The wells of the upper plate with the baudruche side of the feeding membrane at the bottom were disinfected using 70% ethanol and left to dry. Before serum was added to the *in vitro* feeding system, each 10 ml of serum was supplemented with 5 µl gentamycin (Sigma-Aldrich, 10 mg/ml). Six hundred µl serum was added to each well and replaced with fresh serum twice daily.

Serum samples were pre-warmed at 37 °C and subsequently added to the wells of the upper plate. The upper plate was sealed with an ELISA plate cover or Parafilm. The unit was placed in a CO_2_ incubator at 37 °C, 90% RH and 5% CO_2_ (as a feeding stimulus) for 48 h to allow larvae to feed. Feeding was stopped by placing the feeding unit overnight at − 20 °C thus freezing the larvae. The percentage of larvae that were engorged (having an enlarged abdomen of at least 2 times the size of the dorsal shield) was determined visually using a stereomicroscope. The studies were performed blind in that the evaluator had no knowledge about the distribution of the test materials over the plate (see statistical evaluation below).

### Statistical evaluation

In order to identify statistically significant results, samples were tested in six-fold. The samples were allocated to the feeding unit such that they were evenly distributed over the plate. This was done to prevent plate-position effects on feeding. The code was kept secret to the evaluator until after determination of the engorgement rate in each well. From the individual values, the average engorgement rate was calculated. Differences between engorgement rates obtained with different test materials were analyzed for statistical significance using one-way ANOVA (Graphpad Prism 5, Graphpad Software Inc.).

## Results

### Production of antisera against rBm86 and rSUB in calves

Friesian Holstein cattle were vaccinated with rBm86 or rSUB and the collected serum was tested for antigen specificity and antibody titers. Western blot analysis showed that bovine serum against rSUB or rBm86 both were specific to their respective recombinant antigens; the anti-rBm86 antiserum stained a protein at Mw 100kDa, and the anti-rSUB antiserum stained a protein at Mw 25kDa and a protein at Mw 50kDa, which could be a doublet of the Mw 25kDa protein (Fig. 1a, c). Specific antibody levels were subsequently quantified as endpoint titers (cut-off Bmin*2) for each specific antigen using a sandwich ELISA. The antibody endpoint titers were calculated as 2log values; generated anti-rBm86 serum had an endpoint titer of 17.0 (Fig. 1d). Vaccination with rSUB resulted in a 2log antibody end titer of 17.8 (Fig. 1b).

### Immunohistochemistry

**Fig. 2:**
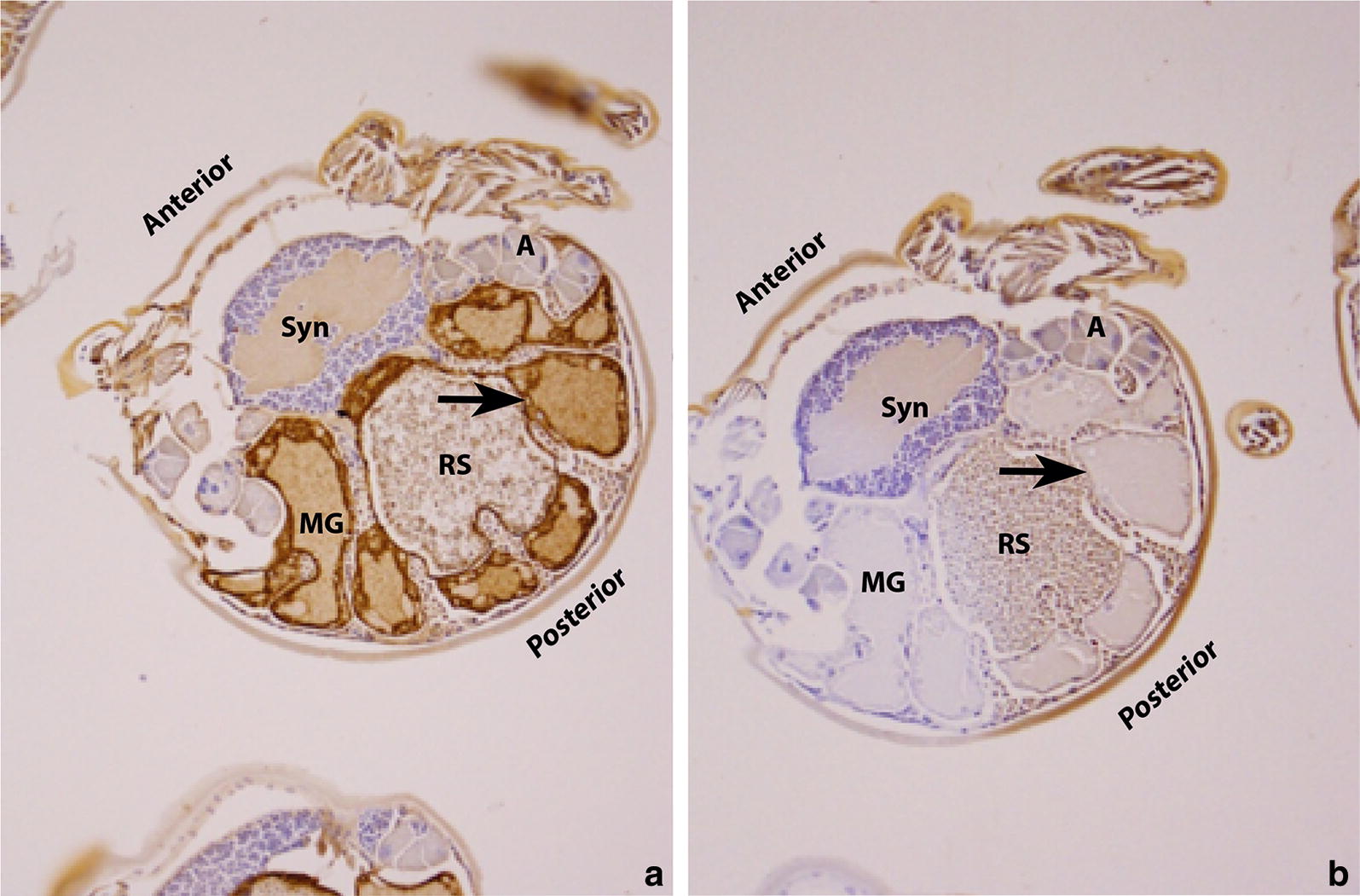
Localization of Bm86 in unfed *R. australis* larvae. Cross sections (20× magnification) of larvae stained with: **a** rabbit anti-Bm86 serum (1:400 diluted) and **b** naïve rabbit serum (1:400 diluted). Sections were pre-treated with proteinase K for 30 min before antibody incubation. Antibodies bound specifically to midgut epithelium (indicated with arrows). *Abbreviations*: Syn, synganglion; MG, midgut; RS, rectal sac; A, acinus

**Fig. 3:**
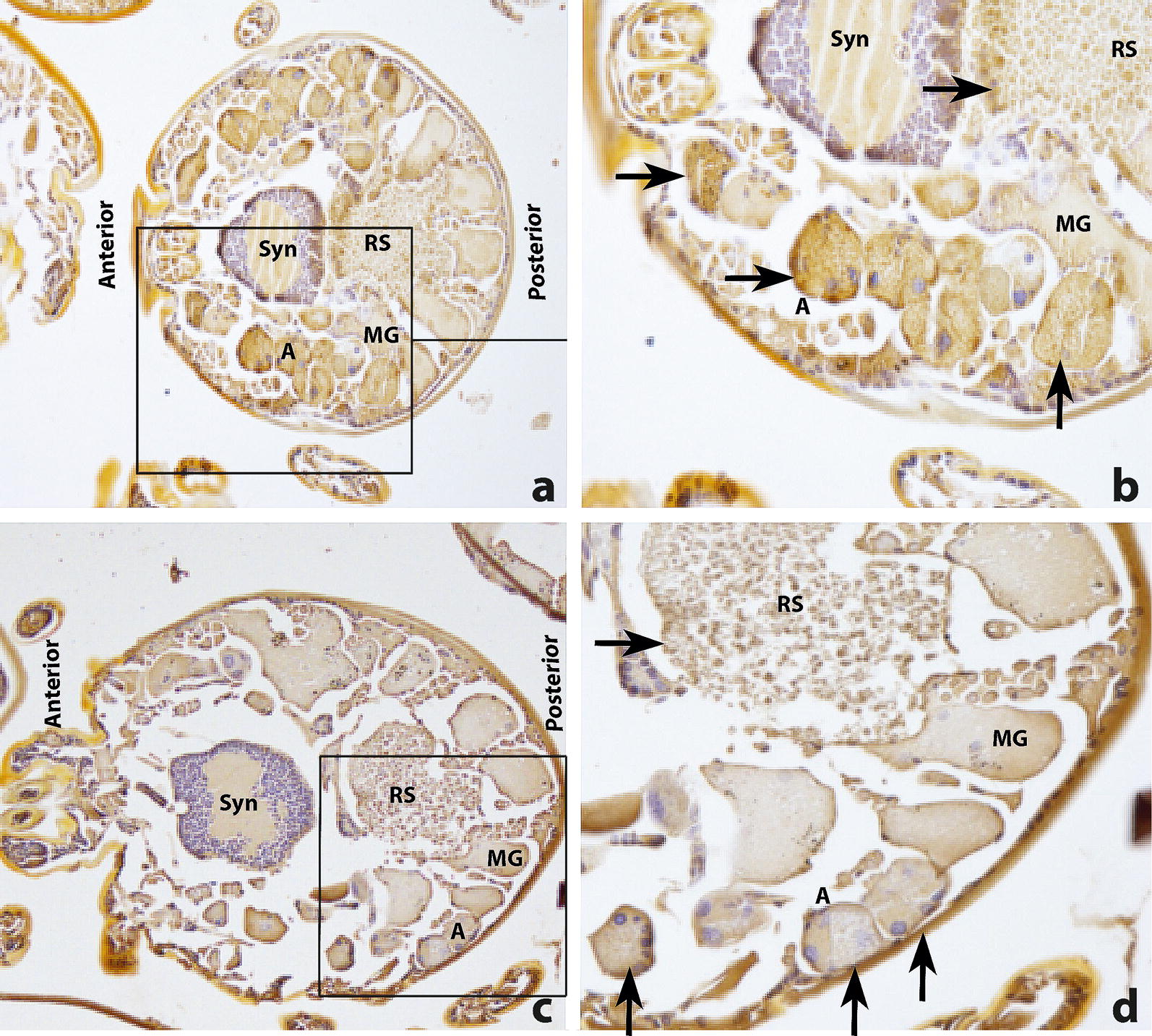
Localization of SUB in unfed *R. australis* larvae with polyclonal anti-SUB rabbit serum. **a** A cross-section (20× magnification) of a larva stained with rabbit anti-SUB serum (1:400 diluted). The square indicates the area depicted in higher magnification (40×) in **b**. IgG showed binding in the acini of the salivary glands, throughout the cytoplasm and within granules (indicated with arrows). **c** A cross section of a larva (20× magnification) stained with naïve rabbit serum (1:400 diluted). The square indicates the area depicted in higher magnification (40×) in **d**. **d** Details of the salivary glands are depicted on the right side (40× magnification). In contrast to the polyclonal anti-SUB serum no IgG binding could be observed in the salivary glands after incubation with naïve rabbit serum. *Abbreviations*: Syn, synganglion; MG, midgut; RS, rectal sac; A, acinus

Immunochemistry was used to visualize which tissues were recognized by antibodies against rBm86 and rSUB. Microscopic slides of whole unfed *R. australis* were incubated with anti-serum directed against each antigen. Antibodies against Bm86 were found to bind specifically to gut epithelium (Fig. 2). Staining of unfed *R. australis* larvae with antibodies directed against subolesin showed that anti-rSUB antibodies bound specifically to the acini of the salivary glands and to the epithelium of the rectal sac (Fig. 3a, c). Reactivity in the acini could be observed throughout the cytoplasm and the globular pattern of staining suggest that the antibodies might bind to small granules within the acini (Fig. 3b, d).

### Effect of anti-rBm86 and anti-rSUB bovine sera on *in vitro* tick feeding

**Fig. 4:**
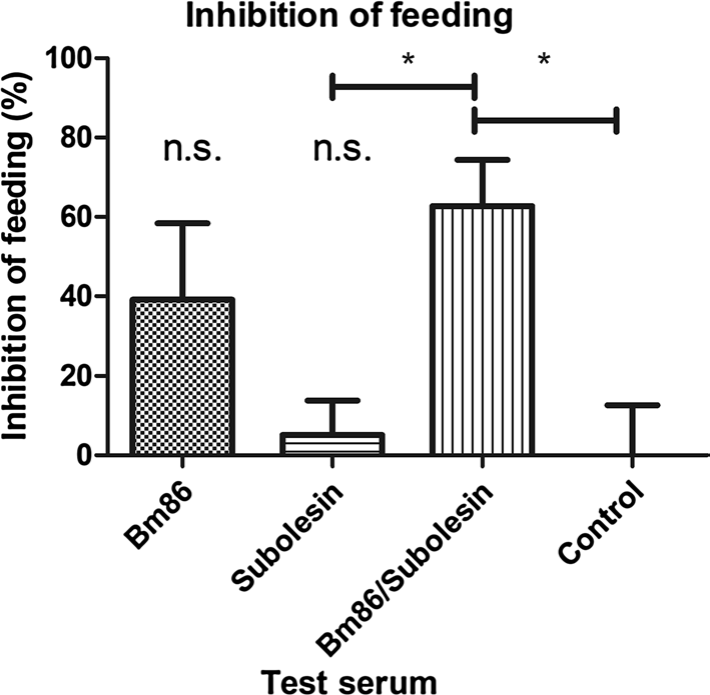
The effect of monospecific antisera against tick antigens and a 1:1 combination of these antisera on *R. australis* larval feeding as compared to tick naïve bovine serum. Larvae of *R. australis* were fed *in vitro* on 600 µl antisera against Bm86 or SUB or on 300 µl anti-Bm86 bovine serum combined with 300 µl anti-SUB bovine serum. After 48 h, ticks were visually scored for feeding. Bars represent the inhibition of feeding as compared to the control group, expressed as a percentage. Error bars represent the standard deviation. *P < 0.05, n.s., not significant

To determine the effect of the monospecific antisera against rBm86 and rSUB on engorgement, antisera were fed *in vitro* to 4–6 week-old *R. australis* larvae in six replicates. Of the larvae that were fed control (undiluted tick naïve bovine) serum, on average 44.3% had fed (data not shown). Feeding of larvae with undiluted antiserum against rSUB did not affect feeding as compared to the control serum (5% reduction; Fig. 4). However, larvae that had been fed with undiluted antiserum against rBm86 exhibited reduced feeding (39% reduction), but this difference did not reach statistical significance. Importantly, when feeding a mixture of equal volumes of serum raised against rBm86 and rSUB (hence each was tested at one time dilution) larval feeding was statistically significantly reduced by 62.7% (*P* = 0.024) compared to control serum (Fig. 4).

**Fig. 5:**
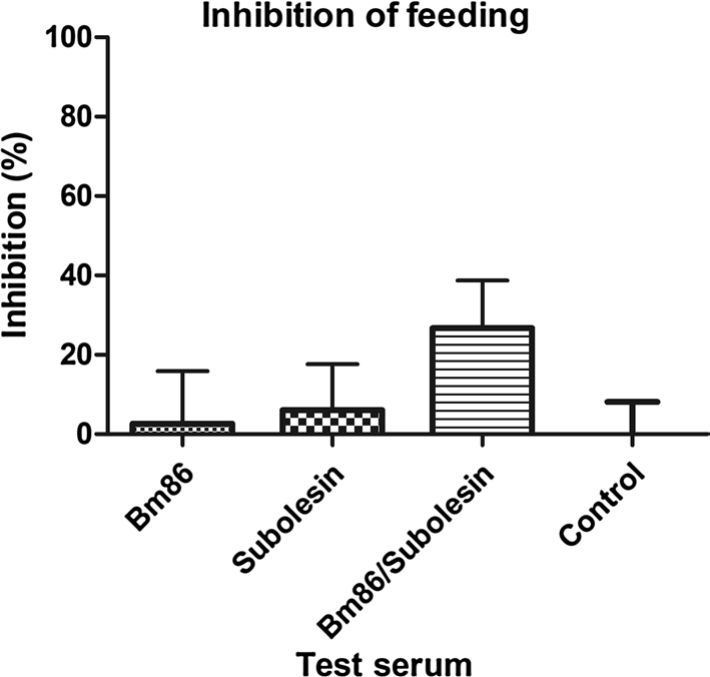
The effect of one-time diluted monospecific antisera against tick antigens and a 1:1 mixture of these antisera on *R. australis* larval feeding as compared to tick naïve bovine serum. Larvae of *R. australis* were fed *in vitro* on 300 µl antisera against Bm86 or SUB diluted with 300 µl naïve bovine serum. Again, 300 µl anti-Bm86 bovine serum was combined with 300 µl anti-SUB bovine serum to assess synergistic effects of both antigens. After 48 h, ticks were visually scored for feeding. Bars represent the inhibition of feeding as compared to the control group, expressed as a percentage. Error bars represent the standard deviation

In order to compare the effect of the two monospecific antisera with the mixture of the two sera at a similar dilution, the monospecific sera were one time diluted with tick naïve bovine serum. The mean feeding of larvae that were fed tick naïve bovine serum was 39.5% (control group). When larvae were fed either one-time diluted anti-rBm86 antiserum or one-time diluted anti-rSUB antiserum, no inhibition of feeding was found (Fig. 5). However, when larvae were fed the mixture of anti-rBm86/anti-rSUB antiserum, feeding was reduced by 26.7%. Although the observed effect was again highest with the mixture of the monospecific antisera, this difference did not reach statistical significance in this experiment (*P* = 0.095).

## Discussion

In search for an improved anti-tick vaccine, the effectiveness of vaccine formulations that contain two or more tick antigens, which have shown partial protection when used as single-antigen vaccines, are being evaluated. The basis of such vaccine is the recombinant *R. microplus* midgut antigen rBm86 that is used in commercially available vaccines. Early experiments in immunized cattle have shown that protection is related to the antibody titer against rBm86 [36, 37], and *in vitro* feeding experiments with adult *R. microplus* showed that serum or purified immunoglobulins (Ig) from immunized cattle reduced the engorgement rate and oviposition in a high percentage of the ticks by damage of the midgut [38]. Similarly, vaccination of cattle with rSUB, a cytoplasmic and nuclear antigen from *R. microplus* ticks, induced partial protection that could be related to the level of anti-rSUB antibodies [39]. Before embarking on vaccination-challenge experiments in cattle to evaluate the efficacy of vaccination against both recombinant antigens, we studied the effect of anti-rBm86 and rSUB antibodies *in vitro* using a recently developed technique that allows feeding *R. australis* larvae with blood and/or serum [35]. Although the assay allows detecting statistically significant differences, variability can occur due to a number of factors such as tick age and condition, and batch differences of membranes and attractants. This can be overcome to some extent by increasing the number of replicates in the feeding assay. The results presented here show that the number of *R. australis* larvae able to feed is reduced when feeding undiluted anti-rBm86 antiserum from immunized cattle compared to tick naïve bovine serum, which is in line with earlier results [35]. When the Bm86 antiserum was diluted once with tick naïve bovine serum the effect on tick feeding was lost. This is reminiscent of the work of Kemp and co-workers, who showed that an increase of anti-Bm86 IgG concentration to a concentration of twice that found in the original serum, increased the level of adult tick damage *in vitro* significantly [38]. Apparently, the effect of anti-Bm86 serum on feeding (and/or gut damage *in vitro*) is an almost a “yes” or “no” effect. In the same paper Kemp et al. show that antibodies can independently induce damage in feeding ticks. As antibodies block endocytosis of fluorescein-labeled BSA by gut cells *in vitro* [5], one might hypothesize that blocking endocytosis adversely affects further engorgement of the larvae. Feeding larvae with undiluted anti-rSUB antiserum did not affect the feeding. Surprisingly, when larvae were fed anti-rBm86 serum that was 1:1 diluted with anti-rSUB antiserum, a 62.7% reduction of feeding was observed that was statistically significant (*P* < 0.05). This suggests that the action of anti-rBm86 antibodies allowed an additional and highly synergistic effect of anti-rSUB antibodies on tick feeding. It could be hypothesized that damage of gut epithelial cells, or lysis, exerted by anti-rBm86 antibodies is a prerequisite for antibodies against rSUB to exert their effect in ticks fed *in vitro*. Alternatively, the effect of anti-rBm86 could be catalyzed by the presence of anti-rSUB antibodies through an as yet unknown mechanism. Any explanation is at this point highly speculative.

In order to determine the localization of the proteins that were recognized by anti-rBm86 and anti-rSUB antibodies, immunohistochemistry was used. Due to the high background upon incubation with a conjugate against bovine Ig (data not shown) we used polyclonal rabbit sera that was raised against the two recombinant proteins instead of the bovine antisera. Antibodies against rBm86 were found to bind specifically to gut epithelium as has been reported previously, and did not stain the acini of the salivary glands [38, 40, 41]. In contrast, antibodies against rSUB reacted with some, but not all, of the acini of the salivary glands of unfed *R. australis* larvae. Reactivity in the acini was observed throughout the cytoplasm. This was unexpected as subolesin, which is a homologue of akirin, is thought to be an intranuclear protein [14, 15]. Consequently, subolesin does not seem to be present only in the nucleus, or the observed reaction outside the nucleus might be explained by cross-reactivity of the anti-rSUB antibodies with an epitope on another protein. However, as the presence of SUB in the cytoplasm of cells in tick salivary glands was described previously for adult *R. microplus* [30], it is most likely that indeed SUB is also present in the cytoplasm of unfed larval salivary glands. Although the exact mechanism for the observed synergistic effect on larval feeding *in vitro* is unknown, it could be related to the different tick tissues targeted by each antibody; in *R. australis* larvae anti-rBm86 antibodies react with the gut epithelium and anti-rSUB antibodies react with the cytoplasm of acini in the salivary glands and with the epithelium of the rectal sac. While it is clear that biological variation can induce differences in effect sizes between *in vitro* assays, the presented *in vitro* reduction of the larval feeding gives high expectations for studying the efficacy of these antigens on *R. australis* infestation *in vivo*. Differences in effect sizes between *in vitro* assays, shows biological variation between tick batches. While feeding success for all control groups was similar, on average 40% of larvae attached and fed, expression levels of the target antigens might differ between ticks. For instance, for Bm86 it is known that expression levels are low in unfed larvae and shows relatively high variation compared to later life stages [42]. Differences in *in vitro* effect size due to biological variation in larvae would therefore likely to be less pronounced in the *in vivo* situation where all three life stages are involved. *In vivo* infestation experiments with *R. australis* and *R. microplus* on cattle span the entire life-cycle of the tick and therefore measures the summed effect of the tested vaccine on larval, nymphal and adult life stages and their respective molting periods. Since larvae are only the first stage of the life-cycle of *R. australis* and *R. microplus* and they only imbibe small amounts of blood compared to nymphal ticks and especially adult ticks, it might be very well possible that the combination of a vaccine targeting both Bm86 and subolesin will have a higher efficacy on these later stages.

## Conclusions

To determine the potential protective effect of vaccines that contain a mixture of tick antigens, a 24-well *in vitro* feeding assay for *R. australis* larvae was used. It was found that feeding a combination of antisera rose against rBm86 and rSUB reduced the proportion of engorged larvae by 62.7% as compared to controls that received tick naïve bovine serum. As a result, a combined vaccine containing rBm86 and rSUB is appears to be a highly promising formula for further *in vivo* testing.

## Data Availability

Data supporting the conclusions of this article are included within the article.
